# 

**Published:** 1917-08

**Authors:** 


### TABLE OF CONTENTS.

Physicians' Lease Committee, Chicago Rotary Club ------129

New Department for Military Orthopedics Organized by Surgeon General of the Army ----------------------131

Pay and Allowances of The Medical Reserve Corps Members and Other Service Conditions ------------133

Rapid Methods of Early Diagnosis of Renal Tuberculosis --137

Comments on Occupation Tuberculosis ------------------143

The Case of Joan of Arc: Was the Maid Insane? ----------147

Superabubic Prostatectomy -----------------------------154

